# Detailed statistical analysis plan for the Danish Palliative Care Trial (DanPaCT)

**DOI:** 10.1186/1745-6215-15-376

**Published:** 2014-09-26

**Authors:** Anna Thit Johnsen, Morten Aagaard Petersen, Christian Gluud, Jane Lindschou, Peter Fayers, Per Sjøgren, Lise Pedersen, Mette Asbjoern Neergaard, Tove Bahn Vejlgaard, Anette Damkier, Jan Bjoern Nielsen, Annette S Strömgren, Irene J Higginson, Mogens Groenvold

**Affiliations:** The Research Unit, Department of Palliative Medicine, Bispebjerg Hospital, Copenhagen University Hospital, Bispebjerg Hospital 20D, Bispebjerg Bakke 23, Copenhagen, NV DK-2400 Denmark; The Copenhagen Trial Unit, Centre for Clinical Intervention Research, Rigshospitalet, Copenhagen University Hospital, Blegdamsvej 9, DK-2100 Copenhagen Ø, Denmark; Institute of Applied Health Sciences, University of Aberdeen Medical School, Foresterhill, Aberdeen, Scotland AB25 2ZD UK; Department of Cancer Research and Molecular Medicine, Faculty of Medicine, Norwegian University of Science and Technology, Postboks 8905, N-7491 Trondheim, Norway; Section of Palliative Medicine, Department of Oncology, Rigshospitalet, Copenhagen University Hospital, Blegdamsvej 9, DK-2100 Copenhagen Ø, Denmark; The Palliative Team, Aarhus University Hospital, Nørrebrogade 44, DK-8000 Århus C, Denmark; Palliative Team Vejle, Vejle Hospital, Kabbeltoft 25, DK-7100 Vejle, Denmark; Palliative Team Fyn, Odense University Hospital, Sdr. Boulevard 29, DK-5000 Odense C, Denmark; Palliative Team Herning, Herning Hospital, Gl. Landevej 61, DK-7400 Herning, Denmark; Department of Oncology, Rigshospitalet, Copenhagen University Hospital, Blegdamsvej 9, DK-2100 Copenhagen Ø, Denmark; King's College London, Cicely Saunders Institute, Department of Palliative Care, Policy and Rehabilitation, London, SE5 9PJ UK; Institute of Public Health, University of Copenhagen, Øster Farimagsgade 5, DK-1014 København K, Denmark

**Keywords:** palliative care, advanced cancer, randomized clinical trial, quality of life, needs assessment, patient satisfaction, cost-effectiveness, data interpretation, statistical analysis plan, protocol

## Abstract

**Background:**

Advanced cancer patients experience considerable symptoms, problems, and needs. Early referral of these patients to specialized palliative care (SPC) could offer improvements. The Danish Palliative Care Trial (DanPaCT) investigates whether patients with metastatic cancer will benefit from being referred to ‘early SPC’. DanPaCT is a multicenter, parallel-group, superiority clinical trial with 1:1 randomization. The planned sample size was 300 patients. The primary data collection for DanPaCT is finished. To prevent outcome reporting bias, selective reporting, and data-driven results, we present a detailed statistical analysis plan (SAP) for DanPaCT here.

**Results:**

This SAP provides detailed descriptions of the statistical analyses of the primary and secondary outcomes in DanPaCT. The primary outcome is the change in the patient’s ‘primary need’. The ‘primary need’ is a patient-individualised outcome representing the score of the symptom or problem that had the highest intensity out of seven at baseline assessed with the European Organisation for Research and Treatment of Cancer Quality of Life Questionnaire (EORTC QLQ-C30). Secondary outcomes are the seven scales that are represented in the primary outcome, but each scale evaluated individually for all patients, and survival. The detailed description includes chosen significance levels, models for multiple imputations, sensitivity analyses and blinding. In addition, we discuss the patient-individualized primary outcome, blinding, missing data, multiplicity and the risk of bias.

**Conclusions:**

Only few trials have investigated the effects of SPC. To our knowledge DanPaCT is the first trial to investigate screening based ‘early SPC’ for patients with metastatic cancer from a broad spectrum of cancer diagnosis.

**Trial registration:**

Clinicaltrials.gov identifier: NCT01348048 (May 2011).

**Electronic supplementary material:**

The online version of this article (doi:10.1186/1745-6215-15-376) contains supplementary material, which is available to authorized users.

## Update

### Introduction

The Danish Palliative Care Trial (DanPaCT) investigates the effects of early specialized palliative care (SPC) for patients with advanced cancer. The trial is a multicenter, parallel-group, superiority clinical trial with 1:1 randomization conducted at six Danish SPC centers. A design and protocol paper describing the design of DanPaCT has previously been published [1].

This paper describes the detailed statistical analysis plan (SAP) for DanPaCT. In accordance with good clinical practice [2] and to avoid outcome reporting bias [3], this SAP was developed before the database was locked and data analysis was initiated. The last patient in the DanPaCT was randomized on 13 December 2013. The last follow-up questionnaire was sent out in March 2014. Data on survival will be retrieved in June 2014. The DanPaCT project group agreed on this SAP on 14 May 2014, and an outline of this SAP was posted on clinicaltrials.gov in June 2014.

### Trial overview

In DanPaCT, consecutive patients from departments of oncology were randomized to standard treatment plus SPC (the experimental group) versus standard treatment (the control group). Inclusion criteria were a) metastatic cancer, b) 18 years or older, c) no prior contact with SPC, and d) a palliative need according to the screening questionnaire. Patients who were eligible according to the inclusion criteria and who consented to participate after having received written and verbal information about the trial were randomized. Patients who were randomized to the experimental group were subsequently referred to the relevant SPC center.

**Figure 1:**
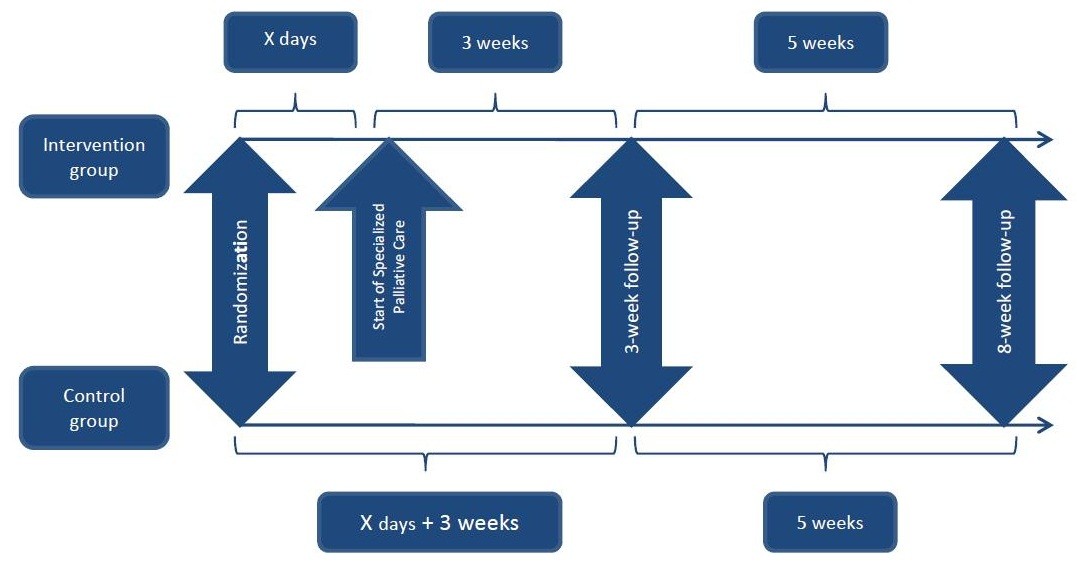
**Time points in the Danish Palliative Care Trial (DanPaCT).**

The screening questionnaire constitutes the entry data (baseline), and patients receive similar questionnaires at the three- and eight-week follow-up. For the experimental group the three-week follow-up was sent three weeks after their first contact with SPC. For the control group it was sent at a matching time. The eight-week follow-up was sent five weeks after the three-week follow-up (see Figure 1). The questionnaires consist of the European Organisation for Research and Treatment of Cancer Quality of Life Questionnaire (EORTC QLQ-C30) [4], a questionnaire measuring the patients’ satisfaction with the health care system, the FAMCARE-P16 scale [5], and the Hospital Anxiety and Depression Scale (HADS) [6]. In addition, data on survival and healthcare costs will be collected. The primary outcome is reduction in the patient’s primary need and secondary outcomes are the seven scales that are represented in the primary outcome, but each scale is evaluated individually for all patients (see sections on primary and secondary outcomes).

The protocol has been approved by the local regional ethics committee (the Ethics Committee for the Capital Region, Denmark; journal number H-3-2010-144) and the Danish Data Protection Agency (journal number BBH-2011-05 DanPaCT) and is registered at http://www.clinicaltrials.gov (NCT01348048).

#### The primary outcome and sample size

The primary outcome is the change in the patient’s primary need. It is estimated as the difference between the experimental and the control group in the change from baseline to the weighted mean of the three- and eight-week follow-up measured as area under the curve (AUC) for the EORTC QLQ-C30 scale score that constitutes the primary need.

The primary need is the one of the following seven scales from the EORTC QLQ-C30 that had the highest score (representing most symptom intensity or most reduced function) at baseline: physical function, role function, emotional function, nausea and vomiting, pain, dyspnea, or lack of appetite. The primary outcome is thus a patient-individualized outcome representing the score on one of seven different scales (for example, patients having the pain-scale as the scale with the highest intensity at baseline will have pain as their primary outcome). If a patient had the same intensity on two or more scales, one of them was randomly assigned as the primary need by the Copenhagen Trial Unit (CTU) based on a simple randomization between the needs.

We know from previous studies that the standard deviation (SD) for a difference between repeated measurements (three weeks apart) in the EORTC QLQ-C30 ranges from 15 for the primary needs with the lowest SD to 22 for the one with the highest SD (unpublished data of patients referred to department of Palliative Medicine, Bispebjerg Hospital collected by MAP, PS, LP, MG from the author list). Therefore, we assume that the primary outcome has an SD of 20 points in the present trial. We wish to be able to detect a minimal relevant difference of 7.5 points (a difference between 5 and 10 is normally judged clinically significant for scales in the EORTC QLQ-C30 [7]). With a risk of type I error of 0.05 for two-sided confidence intervals and type II error of 0.10, we need 150 patients in each intervention group (that is, a total of 300 patients, or about 50 from each of the six centers).

#### Secondary outcomes and power

The secondary outcomes are all of the scales evaluated to determine the primary need (that is, physical function, role function, emotional function, nausea and vomiting, pain, dyspnea, and lack of appetite). Each scale will be analyzed individually for all patients. We calculated the power based on the following premises: a) a sample size of 300 participants (150 in each group); b) a wish to detect a clinically relevant difference on the AUC that corresponds to a difference of 7.5 points on a 0 to 100-point scale; c) an SD expected to be 20 points; d) alpha set to 0.01 (conservative to protect against multiplicity); and e) an expected correlation between baseline and the eight-week follow-up of 0.4, between baseline and the three-week follow-up of 0.6, and between the three- and eight-week follow-up of 0.7. With these premises, we have a power of 83% for each of these secondary outcomes (that is, a risk of type II error of 17%).

Survival is also a secondary outcome. For this power calculation, we used data from the Temel *et al*. trial [8]. Although the Temel *et al*. trial only included lung cancer patients and only included patients who were recently diagnosed with metastatic cancer, it is reasonable to assume similarity between the two trials regarding survival although we include many different metastatic cancer diagnoses. Temel *et al*. found a hazard ratio for death of 1.7 and a median survival in the control group of 8.9 months. Using these figures, and with an actual recruitment time of 30 months (May 2011 to December 2013), a six-month follow-up on survival (as in the present trial), a sample size of 150 patients in each group, and then, with an alpha of 0.01, we will have a power of 88% (that is, a risk of type II error of 12%).

#### Exploratory outcomes

The trial has the following exploratory outcomes:

1. The remaining scales from EORTC QLQ-C30 [4] ( cognitive function, social function, overall quality of life, fatigue, insomnia, constipation, diarrhea, and financial difficulties).
2. Anxiety and depression measured with the Hospital Anxiety and Depression Scale (HADS) [5].
3. Satisfaction with the health care system measured with the FAMCARE-P16 questionnaire [6].
4. Health care costs per week from the start of the trial to minimum three months after the end of the intervention (including at least number of days of hospitalization, ambulatory visits, home visits, emergency visits and general practitioner (GP) visits).

Serious adverse events are defined as hospitalizations and deaths from the time of randomization to the eight-week follow-up. Serious adverse events will be reported in the primary publication. The remaining explorative outcomes are not expected to be part of the primary publication. However, they will all be published in subsequent publications.

#### Descriptive variables

For background variables and variables describing the trial, see Table 1. The distribution of these variables will be given for both intervention groups, but the potential difference between groups will not be significance tested to avoid unnecessary testing [9].

**Table 1:** **Descriptive variables and their definitions**

Variable	Definition	Assessed by/assessed from
*Baseline variables*		
Sex		The Danish Civil Registration System (called CPR in Danish) which depict sex and age
Age		The Danish Civil Registration System (called CPR in Danish) which depict sex and age
Cancer		Project nurse
Receiving treatment (yes/no)	Whether the patient is receiving active anti-neoplastic treatment at the time of randomization	Project nurse
Education		Questionnaire, self-assessment
WHO performance score		Project nurse
Time since diagnosed with cancer stage four	Assessment based on the TNM (tumor, node, metastasis) system. First time it is documented that the patient has stage four cancer. Staging is updated, so a patient diagnosed with stage two and developing distant metastases at a later time is considered as having stage four when the metastases are discovered	Doctors or students with at least a bachelor degree in medicine
Time since diagnosed with primary cancer		Project nurse
*Study descriptive variables*		
Center	There are six centers included in the study	
Primary need	Each patient is randomized for one out of seven potential primary needs (please see article text)	Questionnaire, self-assessment
Use of health care services outside the hospital	Use of for example, psychologist, physiotherapist or other health care services outside the hospital in the eight-week intervention period	Questionnaire, self-assessment
Cross-over	Patients from the control group receiving at least one physical contact with specialized palliative care (SPC) within the study period	Students
Protocol violations	Patients from the intervention group *not* receiving at least one physical contact with the SPC within the study period	Students

We also collect detailed data on the pattern of the experimental groups’ contact with the SPCs: telephone contacts, home-visits, consultations with other professions (psychologists, physiotherapists, social workers, occupational therapists and so forth), multidisciplinary conferences, and description of SPC start. We further collect data on hospitalizations and outpatients’ contacts with the departments of oncology for both groups. In addition, we collect detailed information on the interventions received in the SPCs and in the departments of oncology. Except from a brief description of SPC contacts, these data will not be part of the primary publication.

### Plan of statistical analysis

#### Significance levels

All tests will be two-tailed. For the primary outcome, the risk of type I error is set to 5% (that is, a significance level of *P* <0.05). If the primary outcome is significant, we will calculate the Bayes factor of the primary outcome using the data from the sample size calculation for the trial as described by Jakobsen *et al*. [10]. The exact procedure can be seen in the supplement to the paper by Jakobsen *et al*[10] and the link to the supplement is provided at the end of the reference. A low Bayes factor (for example, less than 0.1) and a low *P* value (for example, less than 0.05) will correspond to a high probability of an intervention effect equivalent to or larger than the clinical relevant effect used in the sample size calculation. If a Bayes factor is calculated ( because we have a significant primary outcome), the results of this calculation will be used to modify our interpretation of the *P* value and conclusion.

If the change in the primary outcome is at least 7.5 points better (on a 0 to 100-point scale) in the experimental group than in the control group, we believe this to be clinically relevant [7, 11, 12]. However, all results from unblinded trials should be critically evaluated for bias, and this should be taken into account when interpreting results [13, 14]. If the experimental group has shorter survival or more adverse events, then this must, of course, also be included in the interpretation of the clinical relevance of SPC.

As we have eight secondary outcomes, we adjust the significance levels to *P* <0.01 to control the familywise (or cumulative) type I error due to multiplicity [2, 15, 16]. We use an interpretation of our *P* values for the secondary outcomes in accordance with the following (if the effect of the intervention is in the expected direction):

1. *P* ≥0.05: The trial results could not demonstrate an effect of the experimental intervention on the secondary outcome.
2. 0.01 < *P* <0.05: The trial results indicate that there may be a positive effect of the experimental intervention on the secondary outcome. However, the indication is not strong.
3. 0.001 < *P* <0.01: The trial results indicate that there may be a positive effect of the experimental intervention on the secondary outcome.
4. *P* <0.001: The trial results strongly indicate that there may be a positive effect of the experimental intervention on the secondary outcome.

The *P* values of the exploratory outcomes will be provided, but it will be made clear that the analyses are exploratory and that we have reported comparisons of one primary and eight secondary outcomes.

#### Analysis of the primary outcome

The primary outcome analysis will be a modified Intention-to-treat (ITT) analysis. Patients who withdrew consent after randomization, who were randomized by mistake and did not fulfil our inclusion criteria, or who were not alive at the time of the follow-ups, will be excluded from the analysis. All exclusions will be shown in the Consolidated Standards of Reporting Trials (CONSORT) flowchart of patient participation.

In the primary outcome analysis we will use non-monotone multiple imputation for nonresponders if there are more than 5% missing outcomes [17]. The imputations are multivariate normal imputations, and we will use SAS statistical software version 9.3. We use the standard SAS procedure for multiple imputations using the fully conditional specification (FCS) statement [18]. In total, we will make 20 different datasets with imputation based on a regression model using predictive mean matching [17, 18]. Predictive mean matching (PMM) will be implemented with ‘regPMM’ having five ‘people’ in the matching pool (which is the default in SAS). In the model, we will include the following variables if they are predictors of the outcome or of having a missing answer (*P* <0.05 in a univariate model and less than 5% missing on the variable in question): the value of the primary outcome in the baseline, the three-week follow-up, and the eight-week follow-up, intervention group (experimental or control), age, sex, The World Health Organizations’ (WHO) performance score, diagnosis, time since diagnoses with metastatic cancer, receiving treatment, and center. It is recommended only to use variables that are (strong) predictors of the outcome or of having missing answers in the model; otherwise, the variables will only introduce noise [16, 19]. When reporting the results, we will, according to guidelines, report on the extent and distribution of missing data [20].

The primary outcome analysis will be a multiple regression adjusted for the stratification variable using a fixed effect [21]. The primary need is used as a group-variable when included as an adjusting covariate (thus, the score on the primary need is not used).

We expect our primary outcome to be normally distributed as differences are often normally distributed [19]. However, if that is not the case, we will try to transform data into a normal distribution, which is often possible. If this is not possible we will use models of other distributions. If this is not possible, we will use nonparametric tests such as the van Elteren [22].In accordance with the design of the trial, some time elapsed from the baseline questionnaire until the patient was randomized and then again, from randomization to the experimental groups’ first contact with SPC (see Figure 1). The timing of the three-week follow-up was dependent on the experimental groups’ first contact with SPC. An equivalent time was added to the control group, so that both groups received the follow-ups at the same time. The median time from randomization to the three- and eight-week follow-up for each group will be used to calculate the AUC for both groups.

#### Sensitivity and explorative analyses of the primary outcome

Sensitivity analyses will be made to test the robustness of the conclusion according to the following rules: a) they should address the same questions as the primary analysis; b) it should be a possibility that they will arrive at another conclusion; and c) if another conclusion is reached, there shall be genuine uncertainty of which one is correct [23]. A description of the types of sensitivity analyses can be seen in Table 2.

**Table 2:** **Description of the sensitivity analyses that will be made for the primary outcome**

	Imputations	Adjusted	Area under the curve (AUC)	Intention-to-treat (ITT) principle
**Fully adjusted analysis**	Yes, as in the primary analysis	Adjusted for covariates (center, WHO performance status, time since the patient was diagnosed with metastases, treatment status, sex, age, diagnosis and education) that are associated with the primary outcome (*P* <0.10 in a univariate test) and where missing <5%	Yes, as in the primary analysis	Modified ITT analysis as in the primary analysis.
**Complete case analysis**	No	Yes, as in the primary analysis	Yes, as in the primary analysis	Violated, no imputations for patients who did not respond to the questionnaire. Analysis may be biased
**Model for repeated measurement analysis**	Yes, as in the primary analysis	Yes, as in the primary analysis	No. The three- and eight-week follow-ups are assessed individually	Modified ITT analysis as in the primary analysis
**Per protocol analysis**	Yes, as in the primary analysis	Yes, as in the primary analysis	Yes, as in the primary analysis	Violated, excluding patients who did not receive the experimental intervention (defined as at least one contact to the SPC team). *The analysis thus only describes the effect of the intervention on those complying with the intervention, which is a subgroup of patients*
**Including patients who died**	Yes, as in the primary analysis	Yes, as in the primary analysis	Yes, as in the primary analysis	ITT analysis. Multiple imputations for patients who do not respond or die

In addition, to test if the effect of SPC is the same regardless of what the primary need is, we will treat the trial as seven different trials - one trial for each need included in the primary outcome - and carry out a random-effects meta-analysis on the results of each primary need. This analysis will be considered an explorative analysis of the primary outcome, and the results of this analysis will be included when discussing the primary outcome of the trial.

#### Analysis of secondary outcomes

The analyses of the seven scales from EORTC QLQ-C30 will use all the same principles as described for the primary outcome including the sensitivity analyses. Survival will be analyzed using a Kaplan-Meier plot. Patients who are alive three months after the end of data-collection will be censored on this date. A Cox regression will be made, adjusted for the stratification variable. A sensitivity analysis will be made, adjusting for the same covariates, with the same criteria as in the first sensitivity analysis of the primary outcome.

#### Exploratory outcomes and subgroup analyses

For serious adverse events, we report the number of hospitalizations and deaths in the eight-week trial period. The analyses of the other exploratory outcomes will not be dealt with in detail here. The overall principles regarding questionnaire data (the remaining scales from EORTC QLQ-C30, HADS and FAMCARE-P16) are that they will be analyzed as complete case analyses and interpreted accordingly. Any subgroup analyses will be exploratory, and it will be stated in the papers that they were *post hoc* subgroup analyses.

### Blinding

#### Patients and questionnaires

Patients could obviously not be blinded to intervention. However, the specific primary outcome of the trial was not revealed to them. In the patient information, it was written that: ‘the aim of the trial is to investigate if it helps people who have reported symptoms and/or problems to be referred to specialized palliative care. In addition it is investigated whether it has consequences for their satisfaction with treatment and care, their survival and healthcare costs’.

All questionnaires were double-entered and compared by students who were not investigators.

#### Register data

Survival will be retrieved from the Danish Civil Registration System (called the CPR-register), and serious adverse events and contacts with the health care system will be retrieved from a Danish Patient Registry (called Landspatientregistret in Danish).

All medical records for the patients have been retrieved for the period from randomization to the eight-week follow-up. The medical records will be split into the medical records from the department of oncology and those from the palliative care team. Hereafter, the medical records will be blinded by students who will delete all paragraphs that can be related to assignment (for example, deleting sentences such as, ‘the patient expressed satisfaction with the palliative care team’). When the medical records have been blinded they will be assigned new identification numbers. Students code the medical records for interventions and contacts while the medical records are in a blinded format.

#### Data-management and analyses

Data management will be done by ATJ. All decisions will be recorded in a log book, and all more vital decisions will be made in consultation with at least two members of the investigator group (one of whom is the principle investigator).

Analyses will be made by MAP, who is blinded to the identity of the two intervention groups, which will be denoted Y and X (or vice versa). Results will be presented blinded in the same way for the investigators, and conclusions regarding the results will be drawn by the investigators and written down while the interventions are still blinded. The blinding will not be broken before all analyses of primary and secondary outcomes have been conducted.

### Discussion

#### The primary outcome: a patient-individualized outcome

Our primary outcome is a patient-individualized outcome. It consists of one of seven different EORTC QLQ-C30 scales, and each patient is only represented with one of the scales. The scale that represents each patient reflects the patient’s main symptom or problem. The great advantage of this primary outcome is that all patients are being assessed for an outcome that is relevant to them. For example, for a patient not having any constipation, improvement in the constipation score cannot be expected even with optimal palliative care. Therefore, this approach prevents the ‘dilution’ of effect arising from inclusion of clinically less relevant measurements (including symptoms/problems the patients do not have).

Clinical trials testing interventions in heterogeneous patient populations may choose to select a single, common symptom (e.g., pain) as the primary outcome but this approach may be problematic if many of the patients do not have that particular symptom. Alternatively, a global quality of life index (a sum of the score on several different symptom/problem scales such as FACT-L [8]) can be used with the expectation that this measures a range of relevant aspects. However, a global index may still be problematic if many of the items measure symptoms/problems that are not present in the patients. In both cases the effect of the intervention may be diluted in the measurement, leading to the risk of false negative results and problems with interpretation.

In relation to specialized palliative care, it is well-known that patients are heterogeneous as they are referred for many different problems, e.g., pain, constipation, depression, anxiety, existential, or psychosocial problems. We cannot expect an effect on all symptoms/problems when the patients only experience some of them. We therefore use a patient individualized outcome in DanPaCT. To further explain the point, consider the following hypothetical example. In a trial two subgroups of patients were included. About half of the patients had arm fractures and the other half had leg fractures. If a primary outcome focusing on arm mobility were used, only half of the patients could be expected to improve. Alternatively, a 'global index' of arm and leg mobility could be used but again this would be introducing noise (and hence dilute effect) because even the arm patients benefiting from the treatment would probably not improve on the items measuring leg mobility. Another approach would be an individualized outcome like the one used in DanPaCT: arm fracture patients were evaluated with the arm mobility score and vice versa. The trial would have one outcome (change in the mobility related to the site of fracture), and hence all patients would be evaluated in a relevant way, thus minimising noise in measurement.

One could also argue that we should have made seven different studies - one for each of the scales in the primary outcome. However, this would be highly expensive and time-consuming. In addition, it is the basic assumption in the present trial that SPC can relieve many different symptoms or problems if they are important for the patient or causing the patient distress, and therefore, it is relevant with a trial investigating the combination of these.

#### The primary outcome and ITT considerations

The primary analysis is a modified ITT analysis as we will exclude those who a) withdrew consent, b) were mistakenly randomized or c) died before the eight-week follow-up assessment. It will be clear from the CONSORT diagram how many patients were excluded for each reason. Only very few patients withdrew consent, and therefore. this is not likely to cause any bias. Likewise only few patients were mistakenly randomized, and they were removed based on objective criteria, regardless of group allocation and no matter what happened after inclusion. When this is the case, it is often considered to be without risk of bias to remove them from the analysis [24].

One can ask whether it is the correct decision to leave out those who died from the primary analysis, as this may be considered a violation to the ITT principle. The main argument for doing so is that it does not make sense to discuss the quality of life of someone who is dead. Nor does it seem much better to speculate about what their symptoms or problems would have been if they had not died or assuming that the imputed score for a dead person could equally apply to a living person.

The consequence of leaving out those who died is that one can argue that our primary analysis investigates the effect of SPC on the primary needs of those who are alive eight weeks after randomization only. However, we argue that it is only relevant to investigate the effect of SPC on the primary need of those who are alive. Or, to argue the other way round, if patients who die shortly after inclusion differ from other patients on certain baseline characteristics (which is not unlikely) and if we include the data from these patients (for whom it did not become relevant) in the estimation of the primary outcome, then such a result would be extremely difficult to interpret clinically. Note, however, that patients who died during the trial are included in a sensitivity analysis.

The risk of leaving out the patients who died is that one may overestimate the effect of the experimental intervention if there is a difference in mortality in the two groups and the experimental intervention group has the highest mortality (it could also be the other way around). We will therefore conduct a sensitivity analysis including the patients who died, and we will consider the risks of serious adverse events when interpreting the primary outcome as described in the section on ‘significance’. If the primary analyses yield results that are very different from the sensitivity analysis, we will include further relevant sensitivity analyses.

#### Missing data due to nonresponse

Trials in palliative care cannot avoid the problems of missing data due to nonresponders. Our basic assumption is that the missing data are missing at random (MAR), and that nonresponse is not related to the effects of the intervention. Our assumption is based on our knowledge from working with this group of patients: there are many reasons for nonresponse that are not related to the effects of intervention. These patients are frequently hospitalized, they may suddenly get more ill, they are frail, they may get problems concentrating and so forth. In addition, the mechanisms causing nonresponse are also likely to be evident from the assessments that are not missing, For example, patients who are nonresponders due to impaired cognitive function are likely to have a worse cognitive function at baseline [25].

#### Number of secondary outcomes and multiplicity

In this trial, we have more secondary outcomes than is often recommended [16]. However, all of them are highly relevant when evaluating SPC. Further, there may be concern about the interpretation of our primary outcome because this outcome has never been used before. We believe that it will enhance the transparency of the trial’s primary outcome to analyze each of the scales represented in the primary outcome for all patients as a secondary outcome analysis.

To adjust for the familywise error, we considered using the Bonferroni or the Holm’s corrections [15]. However, both corrections are too conservative when working with correlated outcomes [16]. To achieve what we believe to be a more fair balance between type I and type II errors, we choose an alpha level of 0.01 for the secondary outcomes. With an alpha of 0.01, nine independent tests (although we are aware that not all of the tests are independent), and no true group difference, then the risk of at least one familywise type I error (to wrongly conclude that there is a difference for at least one secondary outcome) is 9%. The risk of making two or more such type I errors is at a marginal level of 0.3%.

With an alpha of 0.01, nine independent tests, and a true group difference with an effect size of 0.4, the risk of at least one type II error (to wrongly conclude that there is no difference for at least one secondary outcome) is 85%. The risk of making two or more such type II errors is 54%. Hence, with a lower alpha level, these type II errors were deemed too high.

#### Not stratified by center

It is nearly always considered good practice to stratify by center in a multi-center trial, because one will expect that patients from the same center have a higher intraclass coefficient [21]. To reduce the risk of overstratification, we have chosen not to stratify by center in DanPaCT. We believed it to be more important to stratify for primary need as patients having the same primary symptom or problem may be more similar, and the effect of SPC may vary across symptoms or problems.

#### Area under the curve

There are some variations in the actual time points of when the outcomes were measured. For some patients longer times elapsed before they received and filled in the questionnaires than for others. It could be interesting to use real-time data in the weighting of the outcomes, because ‘time’ could be a factor that influences effect. However, if we used the actual number of days elapsing, then the area under the curve (AUC) for two patients having the same symptom burden would be larger for the person who participated in the trial the longest. To prevent this, one could divide the AUC with the number of weeks the patient was participating to get AUC per week. However, as we do not expect the magnitude of effect to be linearly related to length of trial participation [26], we decided to use the same fixed weighting for the three- and eight-week follow-up for all patients.

We also considered whether the AUC should include the time that passed from baseline to randomization. However, as the events happening in this time period have nothing to do with group assignment (the patients were not randomized yet) we decided that the AUC starts at randomization.

#### Changes from the original protocol

We made the following changes: 1) Originally, we had too many secondary outcomes, and therefore several of these have been reduced to exploratory outcomes. This was decided during the process of writing this SAP. 2) We have adjusted the *P* values further to control for the familywise error. 3) In the protocol, we were to test for differences in marital status and comorbidity between the two groups. These data have not been collected and these analyses will, therefore, not be made. 4) In the imputation model, we wrote that we would include cancer stage. However, all patients have stage four, so this is not necessary. We also changed the criteria as to how variables will be chosen for the imputation model. 5) In the Danish version of the protocol, we described a sensitivity analysis that has now been excluded. In that analysis, we would make a worst-case-scenario analysis where all missing data in the intervention group were substituted with the worst possible score, and all missing data in the control group were substituted with the best possible score. Some sensitivity analyses have been added and the covariates included in the first sensitivity analysis have been changed. 6) Several details have been added.

#### Interpretation of the evidence and considerations on implementation in clinical practice

In order to guide potential future implementation in clinical practice, the results of the DanPaCT project should be analyzed in a systematic review including meta-analyses and trial sequential analyses of all relevant randomized clinical trials based on a public protocol for the systematic review [27–31]. Preferably, the analyses should be based on depersonalized individual patient data [27].

## Authors’ original submitted files for images

Below are the links to the authors’ original submitted files for images. Authors’ original file for figure 1 Authors’ original file for figure 2

## Abbreviations

AUC: area under the curve
CONSORT: Consolidated Standards of Reporting Trials
CPR: Danish Civil Registration System
CTU: Copenhagen Trial Unit
DanPaCT: Danish Palliative Care Trial
EORTC QLQ-C30: the European Organisation for Research and Treatment of Cancer Quality of Life Questionnaire
FAMCARE: a questionnaire measuring the patients’ satisfaction with the health care system
FCS: Fully conditional Specification
GP: general practitioner
HADS: the Hospital Anxiety and Depression scale
ITT: Intention-To-Treat
MAR: missing at random
PMM: Predictive Mean Matching
SAP: statistical analysis plan
SD: standard deviation
SPC: specialized palliative care
WHO: World Health Organization.
