# Supplementary figures and images for: Detailed statistical analysis plan for the Danish Palliative Care Trial (DanPaCT)
Source: Trials. 2014 Sep 26;15:376. doi: 10.1186/1745-6215-15-376 (PMC4190470; doi:10.1186/1745-6215-15-376)

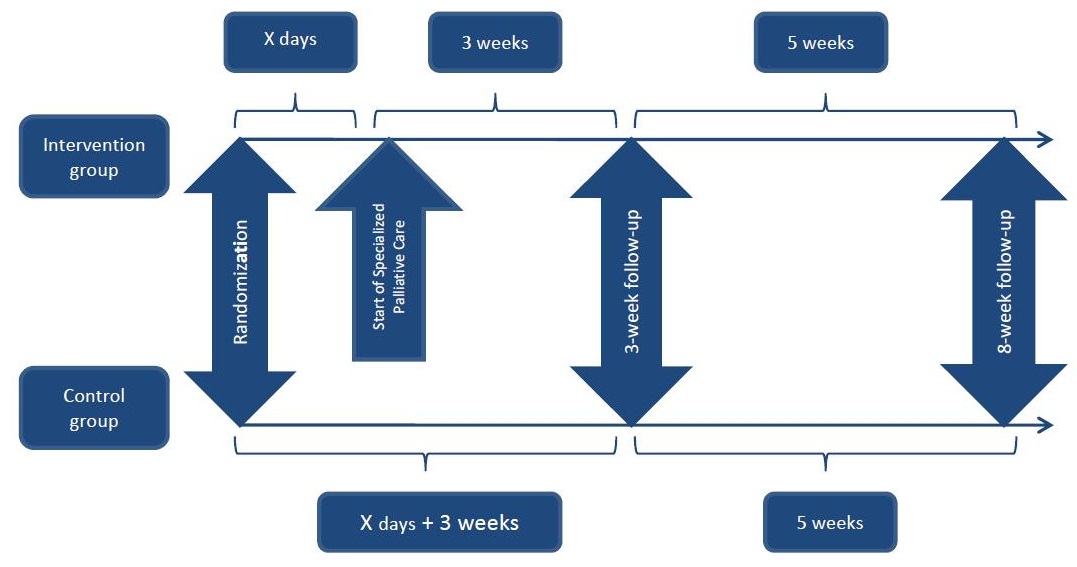

Supplement: Supplementary file 1 — Authors’ original file for figure 1 [file 13063_2014_2245_MOESM1_ESM.jpg]
