# Supplementary material for: Detailed statistical analysis plan for the Danish Palliative Care Trial (DanPaCT)
Source: Trials. 2014 Sep 26;15:376. doi: 10.1186/1745-6215-15-376 (PMC4190470; doi:10.1186/1745-6215-15-376)
Supplement: Supplementary file 2 — Authors’ original file for figure 2 [file 13063_2014_2245_MOESM2_ESM.pdf]

Background
Clinical trials testing interventions in heterogeneous patient populations may choose to select a single, common symptom (e.g., pain) as the primary outcome but this approach may be problematic if many of the patients do not have that particular symptom. Alternatively, a global quality of life index (a sum of the score on several different symptom/problem scales such as FACT-L [8]) can be used with the expectation that this measures a range of relevant aspects. However, a global index may still be problematic if many of the items measure symptoms/problems that are not present in the patients. In both cases the effect of the intervention may be diluted in the measurement, leading to the risk of false negative results and problems with interpretation.

In relation to specialised palliative care (SPC), it is well-known that patients are heterogeneous as they are referred for many different problems, e.g., pain, constipation, depression, anxiety, existential, or psychosocial problems. We cannot expect an effect on all symptoms/problems when the patients only experience some of them. We therefore use a patient individualised outcome in DanPaCT.

Hypothetical example
In a hypothetical trial two subgroups of patients were included. About half of the patients had arm fractures and the other half had leg fractures. If a primary outcome focusing on arm mobility were used, only half of the patients could be expected to improve. Alternatively, a 'global index' of arm and leg mobility could be used but again this would be introducing noise (and hence dilute effect) because even the arm patients benefiting from the treatment would probably not improve on the items measuring leg mobility.

Another approach would be an individualised outcome like the one used in DanPaCT: arm fracture patients were evaluated with the arm mobility score and vice versa. The trial would have one outcome (change in the mobility related to the site of fracture), and hence all patients would be evaluated in a relevant way, thus minimising noise in measurement.
